# Emergency medical services preparedness in mass casualty incidents: A qualitative study

**DOI:** 10.1002/hsr2.1629

**Published:** 2023-10-19

**Authors:** Vahid Saadatmand, Milad Ahmadi Marzaleh, Hamid Reza Abbasi, Mahmoud Reza Peyravi, Nasrin Shokrpour

**Affiliations:** ^1^ Department of Health in Disasters and Emergencies, School of Health Management and Medical Information Sciences Shiraz University of Medical Sciences Shiraz Iran; ^2^ Department of Health in Disasters and Emergencies, School of Health Management and Medical Information Sciences Faculty of Shiraz University of Medical Sciences Shiraz Iran; ^3^ Department of Surgery, School of Medicine Faculty of Shiraz University of Medical Sciences Shiraz Iran; ^4^ English Department Faculty of Paramedical Sciences Shiraz Iran

**Keywords:** disaster, emergency medical services, mass casualty incidents, preparedness

## Abstract

**Background and Aims:**

The effective response of emergency medical services in mass casualty incidents (MCIs) calls for sufficient preparation. The components of preparation must be determined first to achieve this goal. This study aimed to describe the elements of preparedness of emergency medical services for MCIs.

**Methods:**

A qualitative study was carried out on emergency medical service systems in Iran (from April 2022 to mid‐March 2023), using in‐depth semistructured interviews with participants who were managers and members of the incident command team, experts, technicians, paramedics, and telecommunicators of emergency medical services. Interviews were carried out face‐to‐face and via telephone. The data were collected using voice recorder and transcript and analyzed by content analysis method. This study was conducted using the consolidated criteria for reporting qualitative research.

**Results:**

Thirty‐six participants were included in the study. A total of 834 codes were analyzed. Thirteen components were extracted from the study and classified as five categories including “Strengthening management and organization,” “individual and group empowerment,” “capacity expansion,” “technology and infrastructure development,” and “operational response measures.”

**Conclusion:**

Emergency medical service preparedness in response to MCIs is a critical issue. For improving preparedness, the main components must be identified. The study results described the elements of emergency medical service preparedness, which could be used as a framework for developing the national model of emergency medical service preparedness in MCIs.

## Keypoints

- Preparedness is the third element of the disaster management cycle.
- The emergency medical service (EMS) is the frontline of the healthcare system, and improving the EMS preparedness will lead to an effective response to disasters.
- In this study, the main components of EMS preparedness were described, which can be used as a framework for planning in future.

## 1 INTRODUCTION

Management of mass casualty incidents (MCIs) is among the most critical and priority issues of emergency medical service (EMS) throughout the world. MCIs are events that overwhelm the existing healthcare systems and personnel due to many casualties, exceeding local capabilities and resources in a short time. These events typically involve multiple victims who should be managed using day‐to‐day resources (without response to major disasters) and could temporarily affect the local EMS system.^1^, ^2^ Therefore, it is difficult to be completely get prepared to respond to MCIs since EMS and hospital resources are typically fully utilized for routine medical services.^3^ MCIs are diverse depending on their nature, and many disasters such as transportation accidents, terrorist attacks, urban conflicts, mass destruction, and Chemical, Biological, Radioactive, Nuclear and Explosive (CBRNE) incidents are included.^4^

EMS is a critical part of the healthcare system. The most important tasks of EMS in MCIs are incident scene management, triage, life‐saving measures (i.e., airway management, bleeding control), rapid diagnosis, treatment, and transfer of the injured to hospitals. Various studies have highlighted the improvement of EMS readiness in MCIs, such as improving EMS communication, training, coordinating, and organizing the resources.^3^, ^4^, ^5^, ^6^, ^7^

Generally, the response process of EMS to MCIs has many similarities. When the incident message is received by the telecommunication unit, the EMS response process is activated. Depending on the extent of the incident, other organizations such as the police and fire department are also called to the scene. The first teams will assume that the command and the incident command post (ICP) should be established near the staging area. After establishing the safety and security of the scene, EMS teams enter the scene and perform triage, treatment, and transport of the injured to the hospital under the operation command. Simultaneous with the operation, the emergency operation center (EOC) is activated and transmits an emergency notification to the regional EOC. If the dimensions of the incident are large, additional resources will be requested by EOC. In addition, hospitals are also informed by the dispatch unit and activate MCIs response program. The communication process of the operation takes place through a separate communication link with the mobile dispatch in the scene. The operation continues until the last victim is transferred to the hospital. The coordination of the operation scene and hospitals is done through the operation command and central dispatch. The flowchart of EMS response to MCIs in Iran is shown in Supporting Information: Figure 1.

Incidents such as the July 22, 2011 Oslo/Utøya disaster in Norway, school shootings and terrorist attacks in Finland, Sweden, and Denmark, and the shipwrecks of Scandinavian Star and Estonia highlight the importance of EMS preparedness in MCIs. The Great Belt incident in Denmark was accompanied by challenges in MCIs management. In this study, important findings were communication challenges and the consequences of difficult access to the incident site. In addition, it was acknowledged that the Danish EMS system calls for an expansion of capacity in formal education in MCIs management.^8^

Following the terrorist incident in Norway, the combination of four factors was suggested to improve the quality of EMS management in MCIs: (1) major emergency preparedness and competence based on continuous planning, training, and learning; (2) disaster management based on knowledge, trust, and data collection; (3) empowerment through multiprofessional networks; and (4) the ability to improvise based on acquired structure and competence.^9^

As MCIs take place with various natures and call for short‐term management compared to major disasters, it is necessary to formulate a special preparedness framework for MCIs.^10^ First, the components and elements of EMS preparedness must be identified. To this end, this study was conducted to determine the preparedness components of EMS system in MCIs.

## 2 METHODS

### 2.1 Design

This qualitative study was conducted using content analysis approach, which is used to discover the hidden content of qualitative data. Inductive coding was adopted in this approach. Recently, three approaches have been presented in content analysis, including conventional, summative, and directed, which differ in coding and trustworthiness. In the conventional approach, classification codes are extracted directly from the data.^11^ The study adopted conventional content analysis as well. This study was conducted using the consolidated criteria for reporting qualitative research (Supporting Information: File 1).

### 2.2 Setting and participants

Sampling was done from five provinces (Tehran, Fars, Hormozgan, Hamadan, and Kerman) in Iran from April 2022 to mid‐March 2023. The participants were managers and members of the EMS incident command team, EMS field experts, technicians, paramedics, and EMS telecommunicators with rich information who were selected using the purposive sampling method. Sampling continued until data saturation. The inclusion criteria were as follows^1^: having at least a bachelor's degree^2^; having at least 5 years of work experience in various units of EMS^3^; having experiences in MCIs context and being motivated to participate in the study (field experience means that participants have sufficient experience in MCIs context [operations, management, incident command, communications, etc.]. Having sufficient motivation means that the participants should be willing to participate in the in‐depth and follow‐up interview); and^4^ signing an informed consent form.

### 2.3 Data collection

Data were collected through semistructured interviews by the researcher (V. S.), using a pilot‐tested interview guide. The interviews were conducted face‐to‐face, and their follow‐up was mostly carried out using a telephone. The study outline was agreed upon by three members of the research team. Some of the main interview questions were as follows:

(1) In what ways is EMS more vulnerable during MCIs?
(2) What measures are needed to be taken in EMS before MCIs?
(3) What features should EMS systems have to respond to MCIs?
(4) What elements affect EMS readiness in MCIs?

All the interviews were completed and recorded by the main researcher (V. S.). The interviews lasted about 30−90 min, and the participants responded to all the questions according to the research outline. Before the interview, the necessary arrangements were made with the participants. The interviews were conducted in a calm and uninterrupted atmosphere. The researcher refrained from using negative, judgmental, and forgiving statements and attitudes. Data collection continued until saturation, and the researcher stopped sampling when he realized that no new data was obtained and that there were a lot of duplicated data.

### 2.4 Data analysis

A qualitative method was used for data analysis. After each interview, the recordings were transcribed, and the main researcher (V. S.) used the content analysis method to analyze and summarize the data. The steps were^1^: familiarization: the text was read repeatedly to familiarize the subjects with the qualitative data^2^; coding: the open coding method was used to analyze the data line by line, and essential words and phrases (unit meaning) were recorded in the margins of the content; and^3^ integration: each important meaning unit was described in a descriptive code, and codes with the same meaning were integrated.^12^ MAXQDA was used for data organization and coding. After the analysis, two other researchers, who were familiar with the telephone records, carried out a peer review to ensure the validity of the analysis results.

### 2.5 Rigor

Long‐term engagement with participants, member and peer checking, presentation of rich descriptions of data, and data analysis were used for rigor.^13^ Besides long‐term communication with the participants, the research team spent enough time for data collection and follow‐up with the interviewees. Transcriptions along with coding and categories were shown to two qualitative researchers for peer check. Their confirmation was obtained after applying their comments. A brief report of the interviews and the extracted codes were given to the interviewees, who approved it to examine the members. There was also an attempt to consider the maximum diversity in sampling for transferability.

## 3 RESULTS

Thirty‐six participants (24 men and 12 women) were included in the study. All participants entered the study and none of them withdrew from the study. The age of the interviewees was 30−55 years with a mean of 42 ± 0.82. The participants were 14 paramedics, 7 EMS telecommunicators (nurse), and 15 senior managers of the incident command system (6 emergency medicine specialists, 4 medical doctors, and 5 specialists in disasters and emergencies). A total of 834 codes were analyzed. Thirteen components were extracted from the study and classified as five categories including “Strengthening management and organization,” “individual and group empowerment,” “capacity expansion,” “technology and infrastructure development,” and “operational response measures,” as shown in Table 1.

**Table 1:** The characteristics of the interviewees on EMS preparedness in MCIs.

Main category	Subcategory	Participants (%)
Strengthening management and organization	Command and control optimization	32 (89%)
	Compilation of preparedness plan	26 (79%)
	Comprehensive information system	23 (64%)
	Intra and interorganization collaboration	30 (83%)
Individual and group empowerment	Skills enhancement	36 (100%)
	Physical and mental preparedness	25 (70%)
	Promotion of public awareness	19 (53%)
Capacity expansion	Maximum coverage of EMS	22 (61%)
	Logistic and support	28 (78%)
Technology and infrastructure development	Emergency communication system promotion	31 (86%)
	Modernization of EMS systems	17 (47%)
Operational response measures	Improvement of indices time	28 (78%)
	Incident scene operation	31 (86%)

Abbreviations: EMS, emergency medical service; MCIs, mass casualty incidents.

### 3.1 Strengthening management and organization

#### 3.1.1 Command and control optimization

Integrated command is essential in MCIs management. Multiple and scattered commands are the critical challenges in managing these incidents and cause inconsistency, parallel work, disorganization, and waste of time. In the EMS, there are two levels of incident command: (1) central command and (2) operations command. The central command area is at the EMS headquarters or the ICP and is more active in larger incidents where a longer engagement is needed. To the participants, the understanding, field experience, accountability, and strong communication of the command team members play a prominent role in the management of MCIs.

The operation command is the first team member that arrives at the incident scene. All operational teams must have acquired the necessary skills to perform the scene command. One of the suggestions was employment of an EMS operational supervisor and a supervisor in the telecommunication center.

“Using the EMS supervisor has been a very good experience for us. The supervisor facilitates the coordination and operation process.” (P1).

#### 3.1.2 Compilation of preparedness plan

Since the EMS system enjoys a specific framework in daily operations, it must have a written plan for coping with larger incidents. It is better to plan according to the experiences of real events and various regional scenarios and use the consultation and guidance of the elites and experts and common sense.

“In my opinion, the best planning method is to use real everyday experiences and possible scenarios. Theoretical planning is neither reliable nor useful. Experts and elites should participate in planning.” (P2).

#### 3.1.3 Intra‐ and interorganization collaboration

In MCIs, different organizations such as EMS, police, fire, red crescent, and so forth cooperate.

Organizational and interorganizational coordination could be achieved through structural and process reforms, evidence‐based scientific policies, improvement of organizational relationships, and creation of organizational integrity.

“We must enhance the relationship and organizational understanding with the police, fire department, and other organizations. Others must then practice together to reach better coordination.” (P23).

#### 3.1.4 Comprehensive information system

A comprehensive information system helps to enhance EMS preparedness. This information including EMS information involves internal resources such as manpower, equipment, and so forth, and regional information which includes population; geographic characteristics; access roads, rural, and hard‐to‐reach areas; available external resources; number of EMS stations in the areas; Geographic Information System map; risk information; and local capacities.

“We can better define priorities and provide a better response when we have a rich information system of regional risks like traffic accident points and capacities.” (P31).

### 3.2 Individual and group empowerment

#### 3.2.1 Skills enhancement

Although experiences and skills are mostly acquired in daily operations, continuous training, and practice in the field of disasters is critical. The foundation of acquiring skills is laid since studentship.

“Unfortunately, universities now focus more on medical education than disaster management.” (P5).

Training and practice in the MCIs context must be included as a complete package in the annual program of EMS systems. Moreover, it is necessary to conduct continuous group training and exercises. Scenario‐based exercises, real incident reports, and training in MCIs simulated with the participation of related organizations will increase readiness.

“We need to have several operational exercises a year as well as training issues to always be ready.” (P6).

#### 3.2.2 Physical and mental preparedness

MCIs have heartbreaking scenes with a high workload. Thus, all personnel in these operations need to have sufficient physical and mental preparation. EMS students must be screened from the moment they enter universities. Personnel fitness and physical capabilities are critical for the health of the personnel and casualties. For instance, extricating and moving the victims require a lot of physical ability.

Stress management, self‐control, and personnel relationship are essential issues. It is possible to strengthen the psychological aspects of the providers through special educational courses and psychiatric consultations; however, it takes time and experience to reach this goal. Supporting the personnel and their families is another issue.

“When EMS officials called me after stressful missions and gave me peace and encouragement, I did not feel tired.” (P20).

#### 3.2.3 Promotion of public awareness

A major concern in MCIs is the management of local people or bystanders at the incident scenes. Although the law force and other organizations account for providing security and safety, understanding, public knowledge, and community awareness are effective in disaster management.

“In the explosion of one of the religious places, people abnormally placed the injured and even the bodies in ambulances or private cars.” (P3).

However, the spectators' capacity could be used in some interventions that call for general planning and management. Public training via mass media (creating related clips, educational pamphlets, etc.), virtual methods and training volunteer institutions, and so forth are among the ways proposed to promote public awareness.

### 3.3 Capacity expansion

#### 3.3.1 Maximum coverage of EMS

The maximum coverage of EMS could be examined from two perspectives: (1) structural and nonstructural and (2) procedural. From a structural perspective, the number and location of EMS stations must be according to standards like geographical features, risk evaluation, and so forth. From the nonstructural perspective, the most important method was to consider temporary mobile posts in special conditions such as mass gatherings.

“When the peak of some incidents was higher, we set up temporary mobile posts in certain areas; this both improved the speed of operation and covered more issues.” (P2).

From the procedural aspect, the chain dispatch of EMS teams was based on the distance and time to reach the incident site. Local protocols in dispatching methods are important issues. In addition, summoning the EMS personnel and extra‐organizational capacities such as the Red Crescent, army, and other related organizations is used in large incidents.

#### 3.3.2 Logistics and support

In most MCIs, the EMS system can usually respond effectively in terms of human resources and logistics. Usually, EMS stations equip their ambulances according to regional experiences and risks.

“I always fill my ambulance with more equipment and supplies because there are many accidents with injuries here, and the fastest ambulance that arrives after us takes at least half an hour.” (P15).

To the participants, each ambulance must have its own logistic facilities; however, some emphasized the necessity of a regional mobile support system in MCIs. Using ambulance buses and air medical services in MCIs is multipurpose in mobilizing resources and transporting the injured.

### 3.4 Technology and infrastructure development

#### 3.4.1 Emergency communication system promotion

**Figure 1:**
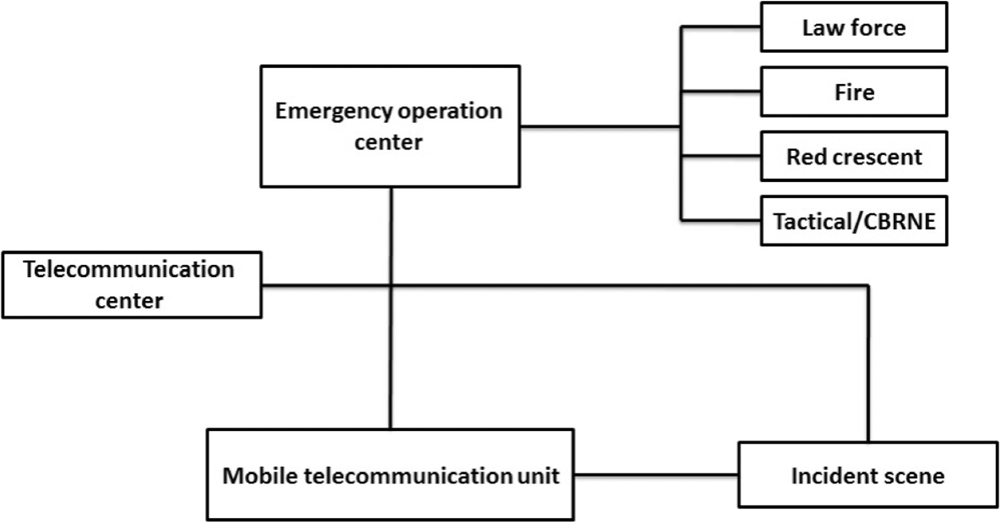
Intra‐ and interorganizational emergency communications. CBRNE, chemical, biological, radioactive, nuclear, and explosive.

Emergency communication is one of the key components in MCIs management. The emergency communication infrastructure is based on optical fiber and radio systems. There are two basic challenges here: their infrastructures are highly vulnerable and they cannot cover nationwide communication. Emergency communication infrastructure must be strengthened by providing digital wireless systems and web‐based communication systems while considering alternative communication systems. The next point is the emergency network communication during the operation. The suggested method is to create a mobile telecommunication center and integration of interorganizational communication system (Figure 1). This method will reduce the communication traffic load and prevent disruptions in the daily operations of EMS.

“It is better to consider a mobile dispatch with relevant organizations in the MCIs. Mobile dispatch eases the communication of the incident scene with the command and telecommunication center.” (P17).

#### 3.4.2 Modernization of EMS systems

Modernizing the EMS system happens in various aspects. Using new educational software, simulation techniques, and training tools to enhance the personnel's skills has a great effect on EMS preparedness. Modern communication systems such as satellite systems, Global Positioning System, new visual and audio‐telecommunication systems, 5G network systems, and so forth enhance EMS communications. Further, using some modern equipment and methods, such as smart glass for estimating the victims, new tools for triage and tracking of the injured, and advanced monitoring systems of EMS operations, is effective in enhancing the operation quality.

“Using the modern technology in all aspects of EMS system improves the quality of emergency response in MCIs.” (P12).

### 3.5 Operational response measures

#### 3.5.1 Improvement of time indices

EMS time indices are considered an important issue in not only MCIs, but also daily operations. These indices include dispatch time, travel time, on‐scene time, transport time, and hospital time. The response time is very important in the prognosis of the injured.

“Time is very important in emergency response situations. Most deaths and complications take place in the initial moments after the accident.” (P23).

**Figure 2:**
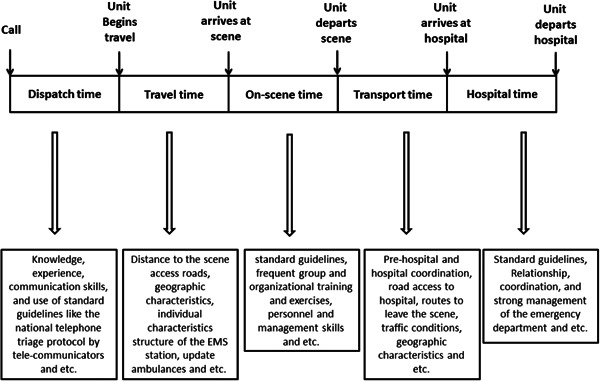
The EMS time indices. EMS, emergency medical service.

First, the time indices should be analyzed and the factors influencing them should be known. These indices and factors affecting them are shown in Figure 2.

#### 3.5.2 Incident scene operation

The operation has several stages including evaluation of the incident scene, triage, treatment, and distribution and transportation. All stages require a high level of EMS preparedness that are achieved through training, practicing, and implementing standard guidelines. However, the EMS systems need to increase activities related to incident scene operation. All stages are displayed in Supporting Information: Table 1.

**Figure 3:**
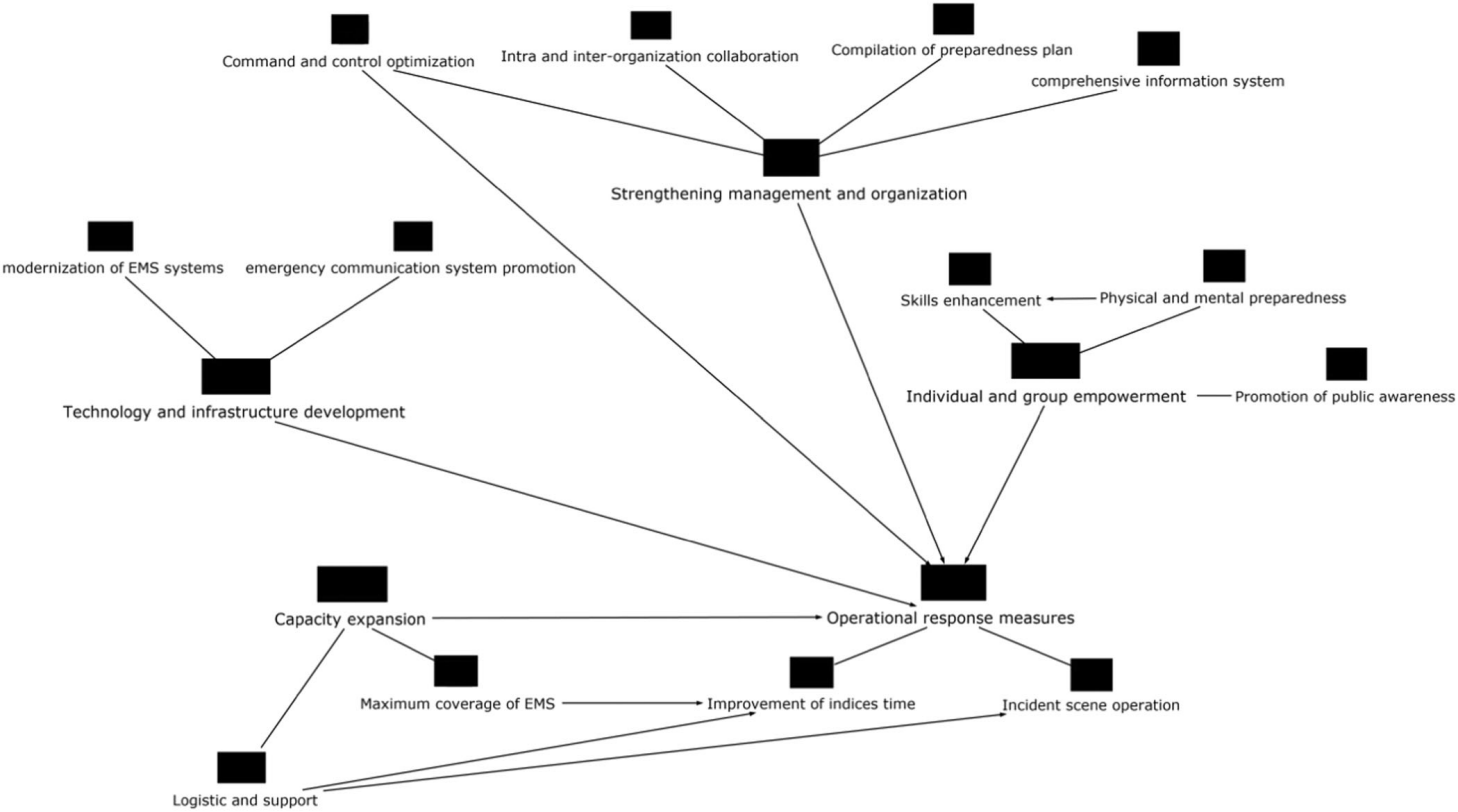
The concept map of EMS preparedness in MCIs. EMS, emergency medical service; MCIs, mass casualty incidents.

There is a conceptual and practical relationship between most of the components, as seen in Figure 3, although the preparedness elements were placed in several separate categories. The general implication of the map is that all preparedness elements are in close relationship to provide a systematic operational response to MCIs.

## 4 DISCUSSION

This study identified the preparedness components of the EMS system in response to MCIs. Thirteen components were extracted from the study and classified into five main categories such as strengthening management and organization, individual and group empowerment, capacity expansion, technology and infrastructure development, and operational response measures.

MCIs call for strong organization and management. Elements like command and control, EMS readiness programs, intra‐organizational and interorganizational cooperation, and comprehensive information systems play an important role in strengthening the EMS response in MCIs. The integrated command is one of the key elements of EMS readiness in MCIs, as affirmed in this study. There is no integrated command system in emergency response system in Iran. In fact, the responding organizations in MCIs each have a separate command and EOC, and the lack of integrity at the scene causes conflict of duties, chaos, and waste of time. The lack of centrality and integrity of the command system is also mentioned as a challenge in Bazeli's study. However, most of the countries in the world, including the United States and neighboring countries such as China, they have an integrated command system. Chongqing (in southern of China) has established a unified 120 dispatching and command system covering the entire city.^1^, ^14^, ^15^ All operational personnel must have adequate competency for command. Although it is common that senior EMS officers should take command on the site of MCIs, in this study different results revealed that all EMS providers should have the ability to command the MCIs at the scene. This result was in the same line with that of the study conducted by Adini et al. who concluded that senior EMS officers should not necessarily act as on‐site MCI commanders.^16^ Some studies have emphasized the formation of quick response teams in some MCIs, but in this study, it was believed that other methods of commanding the incident scene operations by dispatching teams from other places would be a waste of time. Nonetheless, tactical teams take command of operations in some specific MCIs, such as CBRNE.^5^, ^17^

It is essential to develop a special operational plan for MCIs according to possible scenarios and daily experiences of incidents. For all MCIs, a specific operational plan must be developed according to their type. Nevertheless, separate programs have been developed for MCIs in some countries, and the approach of the EMS in response to MCIs is different.^4^, ^6^ In the lesson learned from the report of the real incident in France by Carly, it was also highlighted that preparing and adapting the emergency plans to face this multifaceted threat is crucial.^18^

Several organizations are often involved in MCIs. Law force, fire departments, Red Crescent, specialized organizations of hazardous materials, and so forth are among the partners of the EMS in the management of MCIs. Coordination and collaboration of intra‐ and interorganizations were one of the key components that were extracted from this study.^1^, ^10^, ^19^ This important issue in the study conducted by Sadat in Iran in the field of EMS preparedness in mass casualty traffic accidents was one of the important findings of this qualitative study which was consistent with the findings of the present study.^20^ The findings of the study conducted by Kondo in Japan showed that to provide constant medical assistance at the disaster area, logistic teams, and other disaster medical relief teams must have constant coordination at the medical headquarter command.^21^

EMS systems for managing MCIs call for a comprehensive information system that has complete information inside and outside the system. EMS information system is managed in various ways and used in disasters or everyday situations. In our study, the effectiveness of different methods was not compared. A study conducted by Ruter et al. showed that the on‐line information system was less accurate for storing and retrieving information than a conventional file system. They acknowledged that more technical development was needed before the on‐line system could be recommended for use in major incidents or disasters. However, a comprehensive information system helps the EMS to plan and respond to MCIs.^10^, ^22^

The EMS system must pay special attention to the personnel and group empowerment to effectively respond to MCIs. Strengthening the skills, physical and psychological preparation of EMS personnel, and increasing community awareness were the key components. As to strengthening skills, development and implementation of educational curricula in universities, exercise and training in simulated environments, scenario‐based practices, presentation of case reports and lessons learned, and real experiences are important factors.^8^, ^9^, ^23^ In this study, it was stated that the focus of EMS empowerment is on clinical skills rather than disasters. This empowerment should be a combination of training and standard courses to increase the EMS preparedness in MCIs. In a study conducted by Carenzo et al. in Italy, a variety of standard courses including scene command, triage, regional standards, operational skills, and so forth were held in MCIs context. They described a simple yet interactive simulation and blended‐learning approach, which has yielded good pass rates and good participant satisfaction and involved costs to systematically train emergency medical service personnel.^24^

Given the stressful and busy MCIs environment, EMS personnel must have physical and mental health. Emergency medical students screening upon arrival, continuous monitoring of the physical and mental health status, and motivational support of the personnel and family were among the key elements.^4^, ^25^

One of the challenges in MCIs is the presence of various people and groups that could potentially disturb the focus of the EMS operation. From another perspective, people at the scene of the accident can help as volunteers in some operation processes. The study results indicated that public awareness through public education with various methods was effective in improving public knowledge, awareness, and performance.^9^ In a case report, the impact of the public on MCIs was also mentioned. In this incident, which was related to the overturning of a bus carrying Iraqi pilgrims, some lessons were learned. This case highlights the fact that over‐crowding and laypeople interference at the scene disrupt the relief and rescue. It was suggested in this case that the public education and police monitoring and control should be considered to solve these challenges.^26^

The EMS system is suggested to expand the existing capacities in response to MCIs. The EMS system should expand its existing capacities including manpower and resources, in response to MCIs. The maximum regional coverage of EMS, support, and logistic strengthening of the regional EMS were among the effective components. A burn MCI involving 499 patients occurred at a “color party” in Taiwan on June 27, 2015. In this report, it was pointed out that the collaborative utilization of regional emergency medical services might improve the surge capacity in the field.^27^ Comprehensive coverage of emergency services is possible through structural measures and processes. The support and logistics reinforcement by the internal and external capacities of the EMS is of great importance; however, all ambulances must have sufficient logistic facilities in the EMS.^1^, ^9^, ^27^

The development of EMS technology and infrastructure is critical in all disasters including MCIs. Personnel security, logistic support, and other components of the operation scene management are disrupted when there are communication problems. Besides strengthening the communication infrastructure and related equipment (like radio systems), the telecommunication center and the incident command should consider a mobile telecommunication system in the ICP and specify a separate communication channel to improve communication between the incident scene.^8^, ^28^ A review of medical reports and news articles of mass‐casualty terrorist attacks was performed by Cauwer et al., using PubMed‐archived and nongovernmental reports. In this review, it was expressed that in several cases, communication between different responding actors was poor or nonexisting. Malfunctioning of outdated telecommunication services, inadequate training in the use of communication devices, and unfortunate damage of telecommunication network infrastructure were also worrisome. They concluded that governments should provide sufficient resources to equip hospitals, emergency departments, and ambulance services with back‐up communication systems and invest in training.^29^

The EMS must move along with the progress of technology. Modernizing the EMS system happens in various aspects, such as ambulance, equipment and communication tools, training methods, and operation processes. Some studies have examined the comparative effects of some new methods and emphasized the modernization of EMS systems.^3^, ^30^ Artificial intelligence (AI) is one of the modern technologies that has not yet been developed in the EMS system of Iran. Lu et al. used AI to provide a new technique for the triage of MCIs and more efficient solutions for emergency rescue. They developed an intelligent triage system based on two AI algorithms, namely OpenPose and YOLO. They concluded that the proposed technique may provide an alternative technique for the triage of MCIs, and it is an innovative method in emergency rescue.^31^

The process of EMS operation is of the key elements in MCIs management. Improvement of time indices and the operational management of the incident scene were described as two main components of the emergency operation process. The findings indicated that all activities that resulted in optimal time management in the operation process enhanced the EMS response. Many studies have been carried out on the effect of time indices on the prognosis of injured patients, and almost all of them have emphasized considering effective strategies to enhance these indices.^10^, ^32^

Incident scene operation in MCIs include the incident scene assessment, triage, treatment, transportation, and distribution of the injured. MCIs are usually dynamic scenes; thus, there are many differences in how to manage the scenes.^1^, ^3^, ^16^

The findings showed that the first EMS operational team should conduct the scene assessment. However, in some MCIs such as CBRNE and lack of safety and security, it is necessary to call tactical teams. Operational tactical teams have not yet been developed in Iran. Hughes et al. indicated that by implementing simple yet effective standardized approaches to burn care in MCIs and in supporting the capability of healthcare systems globally to meet and deliver the resources and expertise to manage multiple burn injured patients, patient outcome can be improved and the associated disability‐adjusted life years will be reduced.^5^, ^33^

The injured are triaged after scene evaluation. Using modern equipment in the triage and tracking of the injured as well as the use of experienced personnel were important. There are differences of opinion regarding the treatment of the injured in MCIs. In the American model, the emphasis is on the rapid transfer of the injured to the hospital, whereas in the Franco−German model, it is recommended that one should stay at the scene and take more measures. The study findings indicated that using the two models mentioned depended on the severity of the injured, resources and distance to the hospital, and so forth; it seems that further studies are required to be conducted in this field.^34^

Pediatric and psychological care should be given special attention in all disasters including MCIs.^35^, ^36^ Several unique characteristics of children, elderly people, pregnant women, and other vulnerable groups merit special attention during MCIs because of their age, physiology, and vulnerability. However, there are challenges in the operation process, especially triage, treatment, and transfer of these people to the hospital. These challenges are intensified in the case of lack of resources. Due to the vulnerability of these people and to reduce mortality, special guidelines should be developed for the management of high‐risk and vulnerable groups in MCIs.^37^, ^38^, ^39^

Ethical issues play a prominent role in all aspects of EMS response in MCIs. EMS systems seek to relieve suffering and preserve life, guided by the ethical principles of nonmaleficence (do no harm), beneficence (act for the benefit of others), respect for autonomy (self‐determination), and justice (fairness and equitable allocation of resources). At MCIs, EMS personnel strive to provide the most care to the most people. Ethical considerations in the operation phase, including triage, treatment, and transfer of the injured, are particularly important, especially when resources are limited, and MCIs conditions are complex. Morshedi et al. revealed that the only way to avoid making difficult ethical decisions in MCIs was to either increase resources or avoid MCIs altogether, which is often out of our control.^40^

Distribution and transportation of the injured at the scene of the accident is the last stage of the operation process, which should be carried out with prehospital and hospital coordination. In the EMS system in Iran, there is a system called Medical Care and Monitoring Center, which performs this coordination in close connection with the telecommunication center and the operation command. This system monitors the emergency department of hospitals and coordinates the distribution of the injured according to triage levels. However, the coordination of the distribution of the injured is carried out directly by the telecommunication center in some countries.^41^

Although the EMS system of Iran has many strengths in disaster management, it seems that it is still far from the levels of EMS preparedness in developed countries. Based on the experience, EMS system of Iran has performed acceptable activities in most incidents and disasters.^41^ However, in some important issues, it needs a lot of structural and process strengthening and revision. The most important challenges mentioned in this study are integrated command, interorganizational coordination, integrated training in disaster context, communications, and modern technologies that require many structural and process reforms. In developed countries, these components have significantly progressed. Therefore, it is recommended that the EMS of Iran should benefit from the experiences of other countries and have international cooperations to improve the readiness.

Usually, due to ethical considerations and the dynamic nature of incidents, it is challenging to measure the impact of EMS preparedness components on survival, disability, or mortality. However, each component described in this study is considered as an important benchmark which was expressed by EMS experts. For example, improving the time indicators and the skills of the personnel in triage, treatment and care of the injured, which was obtained from the results of this study, can be effective in the survival of the injured and reduction of mortality. Nonetheless, to measure the impact of the components, studies with a special methodology should be designed to determine the effectiveness of this framework.

The research team attempted to specify the general aspects of EMS preparedness in MCIs. It is suggested that further studies should be conducted in future and describe the aspects of preparedness, specifically in MCIs with different types (CBRNE, terrorism, urban conflicts, mass gatherings, etc.), so that more detailed operational planning could be designed.

## 5 LIMITATIONS

The research team in this study aimed to carry out in‐depth interviews with experienced and key people in EMS systems. It was hard to reach some participants because of the distance and busy schedules. To solve this problem, we made proper communication with the participants, and interviews were conducted with persistent follow‐ups. Moreover, in the EMS system, various groups such as the operation personnel, telecommunication center, managers, and so forth worked with various experiences. A more diverse sampling of various groups was adopted until the saturation of information was reached to this end.

## 6 CONCLUSION

Enhancing the EMS preparedness in response to MCIs is a critical issue. The components of EMS preparedness must be identified to reach this goal. The results of this study indicate that the EMS readiness in MCIs has various aspects which vary according to the existing policies and structures. The main components of EMS preparedness in MCIs were described in the study. The study finding could be used as a framework for developing the national and international model of EMS preparedness in MCIs.

## AUTHOR CONTRIBUTIONS

**Vahid Saadatmand**: Conceptualization; data curation; formal analysis; investigation; methodology; project administration; software; supervision; validation; visualization; writing—original draft; writing—review and editing. **Milad Ahmadi Marzaleh**: Conceptualization; writing—original draft. **Hamid Reza Abbasi**: Formal analysis; investigation. **Mahmoud Reza Peyravi**: Conceptualization; Formal analysis; Methodology. **Nasrin Shokrpour**: Writing—original draft; writing—review and editing.

## CONFLICT OF INTEREST STATEMENT

The authors declare no conflict of interest.

## ETHICS STATEMENT

The study was approved by the ethics committee of Shiraz University of Medical Sciences with the code of IR.SUMS.NUMIMG. REG.1401.020, and consent was obtained from all participants. The subjects were volunteers and were assured that the data would be strictly confidential, and the principle of anonymity would be adhered to. Additionally, the participants could withdraw from the study any time they wished.

## TRANSPARENCY STATEMENT

The lead author Mahmoud Reza Peyravi affirms that this manuscript is an honest, accurate, and transparent account of the study being reported; that no important aspects of the study have been omitted; and that any discrepancies from the study as planned (and, if relevant, registered) have been explained.

## Supporting information


**Supplementary File**: The COREQ checklist (For review not for publishing).

**Supplementary Figure 1**. flowchart of EMS response to MCIs in Iran.

Supporting information.

## Data Availability

The data are not publicly available due to privacy or ethical restrictions, but the data that support the findings of this study are available on reasonable request from the corresponding author.
