# Supplementary material for: Emergency medical services preparedness in mass casualty incidents: A qualitative study
Source: Health Sci Rep. 2023 Oct 19;6(10):e1629. doi: 10.1002/hsr2.1629 (PMC10587387; doi:10.1002/hsr2.1629)
Supplement: Supplementary file 1 — Supplementary File: The COREQ checklist (For review not for publishing). [file HSR2-6-e1629-s001.doc]

**Manuscript:** **Emergency Medical Service preparedness in Mass Casualty Incidents: A qualitative study**

**Supplementary file 1** Consolidated criteria for reporting qualitative studies (COREQ): 32-item checklist

| **No. Item** | **Guide questions/description** | **Reported on Page #** |
| --- | --- | --- |
| **Domain 1: Research team and reﬂexivity** |  |  |
| *Personal Characteristics* |  |  |
| 1. Inter viewer/facilitator | Which author/s conducted the inter view or focus group? | Vahid Saadatmand |
| 2. Credentials | What were the researcher’s credentials? E.g. PhD, MD | PhD, MD |
| 3. Occupation | What was their occupation at the time of the study? | Faculty Member(5) |
| 4. Gender | Was the researcher male or female? | Four male and one Female |
| 5. Experience and training | What experience or training did the researcher have? | Qualitative research methodology, software education and training(MAXQDA), interview method, communication skills |
| *Relationship with participants* |  |  |
| 6. Relationship established | Was a relationship established prior to study commencement? | Yes |
| 7. Participant knowledge of the interviewer | What did the participants know about the researcher? e.g. personal goals, reasons for doing the research | In the first relation with the participants, we explained the objectives and the necessity of this research. |
| 8. Interviewer characteristics | What characteristics were reported about the interviewer/facilitator? e.g. Bias, assumptions, reasons and interests in the research topic | The work experience of the interviewers was the most important characteristics that might influence the interviews. However, bracketing was observed. |

**Supplementary Table 1** Consolidated criteria for reporting qualitative studies (COREQ): 32-item checklist (continue)

| **Domain 2: study design** |  |  |
| --- | --- | --- |
| *Theoretical framework* |  |  |
| 9. Methodological orientation and Theory | What methodological orientation was stated to underpin the study? e.g. grounded theory, discourse analysis, ethnography, phenomenology, content analysis | content analysis |
| *Participant selection* |  |  |
| 10. Sampling | How were participants selected? e.g. purposive, convenience, consecutive, snowball | Purposive sampling |
| 11. Method of approach | How were participants approached? e.g. face-to-face, telephone, mail, email | All methods were face-to-face, but due to social distancing telephone call and virtual methods (whats app) were also used. |
| 12. Sample size | How many participants were in the study? | 36 participants |
| 13. Non-participation | How many people refused to participate or dropped out? Reasons? | None |
| *Setting* |  |  |
| 14. Setting of data collection | Where was the data collected? e.g. home, clinic, workplace | EMS systems in Iran |
| 15. Presence of non-participants | Was anyone else present besides the participants and researchers? | There was not anyone. |

**Supplementary Table 1** Consolidated criteria for reporting qualitative studies (COREQ): 32-item checklist (continue)

| 16. Description of sample | What are the important characteristics of the sample? e.g. demographic data, date | | Page 7, in the result section of the manuscript are described. |
| --- | --- | --- | --- |
| *Data collection* |  | |  |
| 17. Interview guide | Were questions, prompts, guides provided by the authors? Was it pilot tested? | | Data were collected through in-depth semi-structured interviews using a pilot-tested interview guide by the first author. |
| 18. Repeat interviews | Were repeat inter views carried out? If yes, how many? | | No |
|  | |  |  |
| 19. Audio/visual recording | Did the research use audio or visual recording to collect the data? | | The voice recorder of the android system on mobile phone was used. |
| 20. Field notes | Were ﬁeld notes made during and/or after the interview or focus group? | | Yes |
| 21. Duration | What was the duration of the interviews or focus group? | | 30-90 minutes |
| 22. Data saturation | Was data saturation discussed? | | Data saturation was discussed by the research team in several sessions. |
| 23. Transcripts returned | Were transcripts returned to participants for comment and/or correction? | | Yes (page 7 in rigor section) |
| **Domain 3: analysis and ﬁndings** |  | |  |
| *Data analysis* |  | |  |
| 24. Number of data coders | How many data coders coded the data? | | 834 |

**Supplementary Table 1** Consolidated criteria for reporting qualitative studies (COREQ): 32-item checklist (continue)

| 25. Description of the coding tree | Did authors provide a description of the coding tree? | The first three authors provide a description of the coding tree. |
| --- | --- | --- |
| 26. Derivation of themes | Were themes identiﬁed in advance or derived from the data? | The themes derived from the data. |
| 27. Software | What software, if applicable, was used to manage the data? | MAXQDA |
| 28. Participant checking | Did participants provide feedback on the ﬁndings? | Yes |
| *Reporting* |  |  |
| 29. Quotations presented | Were participant quotations presented to illustrate the themes/ﬁndings? Was each quotation identiﬁed? e.g. participant number | The participant quotations were presented in the result section. |
| 30. Data and ﬁndings consistent | Was there consistency between the data presented and the ﬁndings? | Yes, there was. |
| 31. Clarity of major themes | Were major themes clearly presented in the ﬁndings? | Yes, they were. |
| 32. Clarity of minor themes | Is there a description of diverse cases or discussion of minor themes? | Yes. It is described in the result and discussion section of the manuscript. |
