# Supplementary figures and images for: Emergency medical services preparedness in mass casualty incidents: A qualitative study
Source: Health Sci Rep. 2023 Oct 19;6(10):e1629. doi: 10.1002/hsr2.1629 (PMC10587387; doi:10.1002/hsr2.1629)

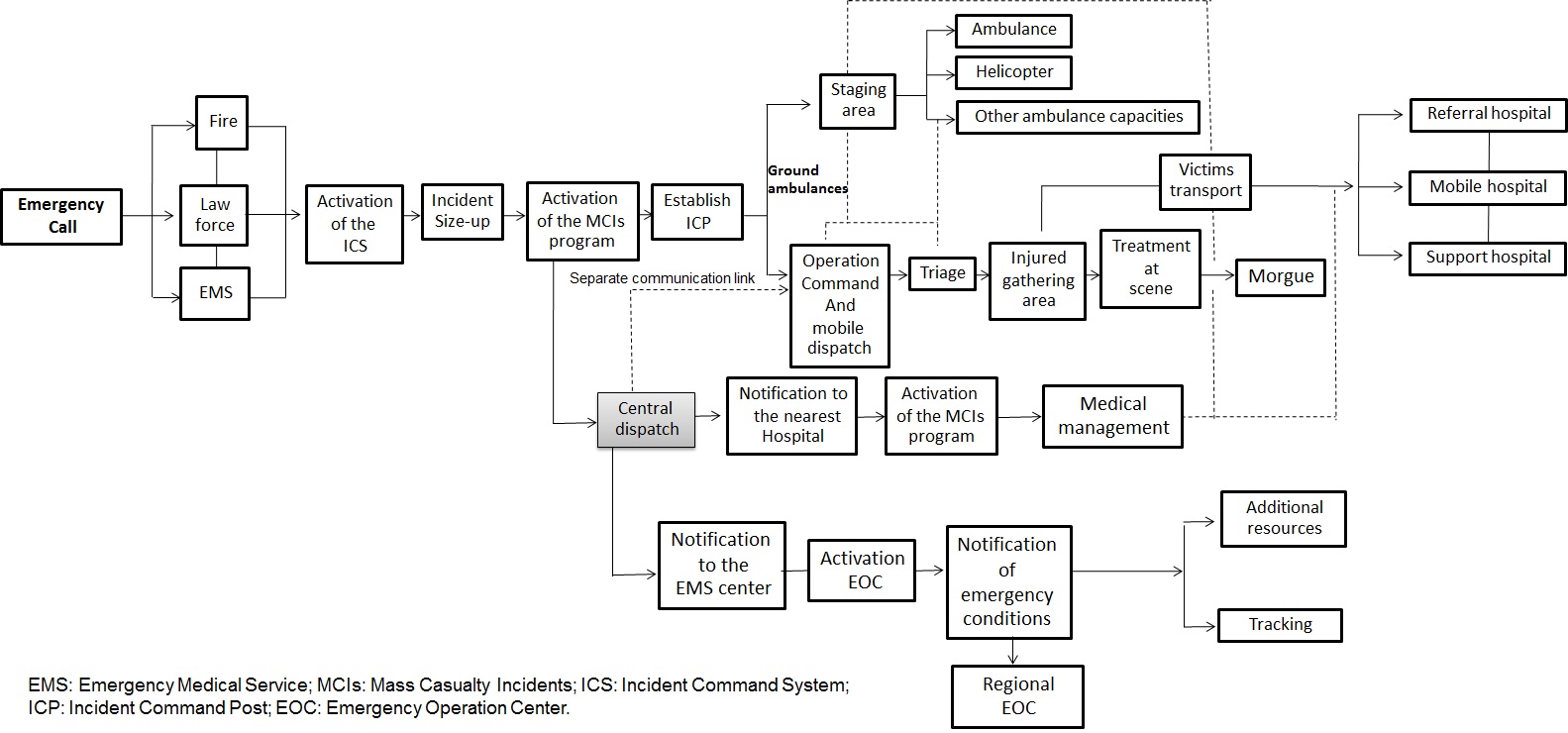

Supplement: Supplementary file 2 — Supplementary Figure 1. flowchart of EMS response to MCIs in Iran. [file HSR2-6-e1629-s002.jpg]
