# Supplementary material for: Emergency medical services preparedness in mass casualty incidents: A qualitative study
Source: Health Sci Rep. 2023 Oct 19;6(10):e1629. doi: 10.1002/hsr2.1629 (PMC10587387; doi:10.1002/hsr2.1629)
Supplement: Supplementary file 3 — Supporting information. [file HSR2-6-e1629-s003.docx]

**Supplementary Table 1.** Elements of incident scene operation.

| **Quotation** | **Description** | **Element** |
| --- | --- | --- |
| “If I want to name the key phase of the operation, evaluating the incident scene is the most important one and requires high professional performance and intelligence.” (P35) | It is carried out by the first team that arrives at the scene. This phase is so important, as it calls for high knowledge, skill, and experience in quickly and accurately collecting information from the incident scene and reporting to the telecommunication unit and headquarters command. Here, deploying operational personnel at EMS stations is critical as MCIs could take place in remote locations where additional resources are unavailable. Hence, operation personnel in EMS stations must be properly arranged in all work shifts, and experienced staff must be present in each EMS station and shift. Moreover, the scenes of MCIs could be unsafe and access to safety and security teams might be difficult, so the technician or paramedic must perform assessments correctly and quickly in unstable conditions. | Evaluating the incident Scene |
| - | Prioritizing patients according to the deterioration of their condition and considering facilities and resources and using local protocols are important in triage. In Iran, START (simple triage and rapid transport) protocol (for injured people over 8 years of age) and jump START (for injured people under 8) are used. However, what was important to the participants as to the triage issue was using experienced and skilled personnel and new technologies in triage, such as smart glasses, bracelets containing barcode readers and accurate tracking capabilities. | Triage |
| “I think treatment to be done at the scene differs for various victims. For instance, I have to drain the pleural space for an injured person who is suspected of tension pneumothorax.” (P3) | The extent to which the injured are treated is different. Most of the participants argued that the patient must be transferred to the hospital immediately. Some stated that in some situations like the distance to hospitals, heavy traffic and blockage of access roads to the hospital, and the case of life-threatening problems, it is better to stay at the scene and perform more interventions. However, the majority believed in performing life-saving measures, primary stabilization, and rapid transfer to medical centers. Moreover, in the treatment area, children and psychological care must be given special attention and standard guidelines need to be used. | Treatment |
| “It is critical that the transport officer has the necessary coordination with the MCMC center and telecommunication for the distribution and transfer of the injured.” (P10) | Distribution and transportation: Prehospital and hospital coordination is very important in the transfer of the injured. There is a unit called Medical Care Monitoring Center (MCMC) that manages prehospital and hospital coordination in the EMS system in Iran. This unit is closely associated with the telecommunication center, and besides hospital coordination with updated equipment, it monitors the emergency departments of hospitals. There should be a medical supervisor at the scene of the accident to monitor the triage, treatment, and transportation of the injured. | Distribution and transportation |
|  | | |
